# In Private Practice: Reflections by a Late Assistant

**Published:** 1912-09-14

**Authors:** 


### IN PRIVATE PRACTICE.

### Reflections by a Late Assistant.

The spheres of the parson and the doctor meet most often at the bedside of a dying patient. But there is another time when the two will each have to decide what to do, and when it may be most advantageous for them to act in harmony. We refer to the time when a patient is found to have an incurable disease which is not immediately fatal, but where the doctor knows that in all probability death is certain within a few months. This question then arises: Shall the patient and his friends be told, or shall they be kept in ignorance of the prognosis? We know no more difficult decision for a practitioner to make. The ultimate responsibility rests, no doubt, upon the doctor, but it is here that harmonious action between clergyman and medical adviser may help, or a difference of opinion stubbornly maintained may cause much trouble. We may lay it down as a principle that every case must be judged on its own merits. And it is here that the man with a wide knowledge of human nature will succeed, where the brilliant fool will fail. The first essential to success as a doctor is a sound knowledge of medico-chirurgical principles; the second a minute attention to detail; the third the power to know your patient. A high development of the last may make up for some deficiency in the first two, and place its owner far above one intellectually his superior, but with a narrow knowledge of human beings. The latter may think it a duty to tell patients straight out that death faces them, or he may take the easier line of calming all fears, true to the principle that while there is life there is hope.

A Parson's Criticisms.

We find that the parson is rather prone to take up the latter stand. Two examples may show our point. A middle-aged bachelor clergyman of the Church of England, with a comfortable income and a wealthy congregation, once criticised with much stringency a doctor who had told another parson that he had but two years to live. Eighteen months had gone, the man was better than he had been for years. His daughter was married, and so he had no anxiety on that score; his money matters had been put right, and he might look forward to many years of useful life. "That doctor," said he, "committed a grave mistake in telling him he had but two years to live. A man's affairs should all be in order so that he is ready to die at any moment. If I were to die to-night everything would be found prepared, so that there would be no hitch." But in this theorising on what should be, he forgot, or did not realise, that the man of whom he spoke was one of those lovable strong weak men, who would never fear to look death in the face, but who, until he faced it, would never see more than a few days ahead. But the doctor had seen that, and the patient faced death and put his affairs in order, so as not to leave his wife with a tangle on her hands, and pushed on his daughter's marriage. Then he seemed to be better, but he died within the two years, some two months after the parson's criticisms.

In the other case a man, who had done much hard work in a poor district in South London, said that he nearly always advocated not telling the patient. He based this on the fact that a medical man can never tell with absolute certainty when a patient will die. He instanced a parishioner of his who had gone into a hospital in 1898. A laparotomy had been performed, and inoperable malignant disease found. The house-surgeon had asked the parson to tell the patient that he had but a few months to live, and, on the parson advising against, had told the patient himself. That man is alive to-day. Everyone knows of such cases where the surgeon has opened an abdomen, found, as he thought, inoperable malignant disease, and been wrong. But how few are these cases compared with those of exploratory laparotomy where the surgeon diagnoses right?

We affirm that he will make fewest mistakes who judges each case on its merits, neither shunning the unpleasant task when it is necessary, nor causing mental pain to a fearful patient by telling unnecessarily. This mental pain may, however, be as great from suspense as from certainty. The fear of death is an innate instinct in the human being, but when death is certain the will can triumph over the instinct, and to the strong man knowledge is less terrible than an uncertain suspicion.

"Please, Sir! will you come at once? The missus is rolling about on the bed in an agony of pain, and is vomiting so that there can't be anything left inside her." "Ah!" thinks the young assistant, "the case of perforated gastric ulcer that I have dreamed of operating on in a country cottage ever since I was a clerk!" "When did you last kill a pig?" says the elderly country doctor. "Why, we've been killing one to-day." "And had the pluck for supper?" "Yes, so we did." "Give her this dose (it's castor oil with some tincture of opium in it) and I'll come in the morning."

A Linseed Poultice.

She was the oldest inhabitant; he the young assistant, fresh from hospital, and full of surgery, but ignorant of country folk and their ways. She had a cellulitis of the dorsum of her foot, which he promptly incised and neatly fomented in approved out-patient style. Then he drove back dreaming of gangrene and amputations, and how to be efficiently aseptic in a two-room cottage. The next day he returned to learn that he had earned the name of "T' Young Butcher," and the same fomentation was still on. Further instructions and a supply of dressings were left. Next day one wringing-wet change of fomentation had been put on, and the skin slough was extending. Dreams of amputation became more vivid. But next morning the chief found the young assistant taking more wool and boric lint, and, hearing it was for old Bessie Barnes with the septic foot, told him not to waste dressings on one that did not understand them, but to give her a bottle of carbolic oil and add it to a linseed poultice. "Those beastly poultices," rang in the ears of the young assistant as he drove out that morning. A week in practice and had he come to this already? But Bessie Barnes was delighted. And next day there was no pus, and the day after the skin edge showed signs of healing, and after the next day it was only necessary to dress it on alternate days. And an eight-mile drive was saved. They did not understand the fomentations, they had no faith in them, nor the skill to put them on hot and dry, nor the time and help to change them often. But every septic focus in the village for centuries had had a poultice on. They put them on hot, and laughed when the patient winced, whereas they took the fomentation off and cooled it. There is scope still for the poultice, and even Lister could not ring its knell. It is cheap, it is easy to apply hot, it holds the heat better than a fomentation. In other words, it is efficient, and it has a place on the bodies and in the beliefs of the people.

A book that I can heartily commend to my fellow practitioners is Mr. Barry Pain's "The One Before." It treats of the husband who is continually interfering and trying to improve the domestic arrangements of the household. I do not mean to advocate a similar interference on the part of all medical men—they would probably be forbidden to subscribe to The Hospital next year! But I would point out that the knowledge requisite for such interference is a necessary part of the doctor's training, particularly when he practises among the poor and middle classes. He should know the difference in price between butter and margarine, and how expensive bacon is. All will be found most excellently put in Dr. Robert Hutchison's "Food and the Principles of Dietetics," but Mr. Barry Pain is more amusing.

